# Abnormal localized [^18^F]FDG accumulation in a Hoffman 3D brain phantom caused by *Pseudomonas aeruginosa* and *Stenotrophomonas maltophilia*

**DOI:** 10.1007/s00259-024-06816-5

**Published:** 2024-07-10

**Authors:** Takashi Kamiya, Sadahiro Naka, Tadashi Watabe, Keiko Matsunaga, Kayako Isohashi, Hidetaka Sasaki, Keita Okamura, Kana Iwao, Isamu Yabata, Mitsuaki Tatsumi, Hiroki Kato, Koichi Fujino

**Figure 1:**
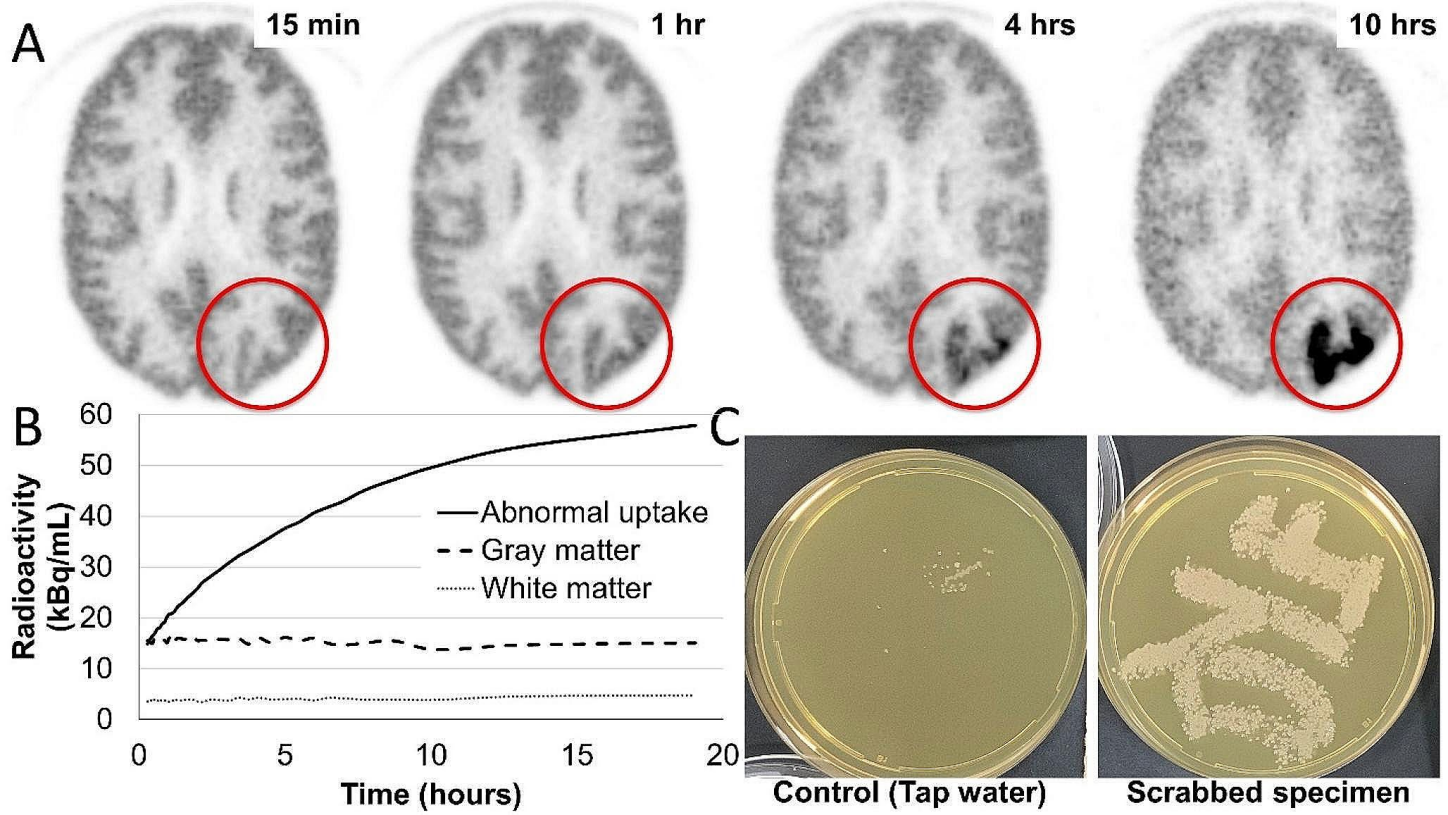
**Fig. 1**

**Affiliations:** 1 Department of Medical Technology, Osaka University Hospital, Suita, Japan Osaka 2-15, Yamadaoka,; 2 Department of Pharmaceuticals, Osaka University Hospital, Suita, Japan Osaka 2-15, Yamadaoka,; 3 Department of Radiology, Graduate School of Medicine, Osaka University, 2-2, Yamadaoka, Suita, Osaka Japan; 4 Department of Advanced Radioisotope Medicine, Institute for Radiation Sciences, Osaka University, Suita, Japan Osaka 2-4, Yamadaoka,

Hoffman 3D brain phantom was scanned to standardize the image quality in a clinical trial. Dynamic [^18^F]fluorodeoxyglucose positron emission tomography ([^18^F]FDG PET), which started 15 min after being filled with [^18^F]FDG (25 MBq), revealed an abnormally increasing accumulation of [^18^F]FDG in the left occipital cortex region at one hour. This abnormal accumulation showed an increasing trend (Fig. 1A, B). The presence of bacteria was suggested by the culture of scrabbed samples taken from the corresponding region in the phantom, later confirmed to be *Pseudomonas aeruginosa* and *Stenotrophomonas maltophilia* (Fig. 1C).

*Pseudomonas aeruginosa* is an aerobic Gram-negative rod with a single flagellum at one end. It is a facultative anaerobic bacterium that generates ATP (adenosine triphosphate) necessary for growth by aerobic respiration and grows by oxidative degradation of glucose in the presence of oxygen [1]. *Stenotrophomonas maltophilia* is a non-fermentative Gram-negative bacterium that consumes proteins and peptides rather than sugars and carbohydrates as carbon and nutrient sources [2]. There have been several reports on the uptakes of [^18^F]FDG [3, 4]. We concluded that the abnormal accumulation was caused by FDG-avid bacterium, *Pseudomonas aeruginosa* and *Stenotrophomonas maltophilia.*

It is preferable to use degassed tap water rather than purified or distilled water to prevent the growth of bacteria. Hoffman 3D brain phantom should be disassembled and completely dried after the scan, especially for multicenter clinical trials.
